# Dry synovitis, RF-negative: a rare entity distinct from juvenile idiopathic arthritis

**DOI:** 10.1093/rap/rkaf144

**Published:** 2025-12-06

**Authors:** Raghad Dghaish, Mahmoud Qouqas, Anas Manhal, Jamal Abdallah, Anas Nairoukh, Basel Zaben, Fawzy Abunejma

**Affiliations:** Faculty of Medicine, Palestine Polytechnic University, Hebron, Palestine; Faculty of Medicine, Hebron University, Hebron, Palestine; Faculty of Medicine, Hebron University, Hebron, Palestine; Faculty of Medicine, Hebron University, Hebron, Palestine; Paediatric Department, Palestinian Red Crescent Hospital, Hebron, Palestine; Faculty of Medicine, Al Quds University, Jerusalem, Palestine; Faculty of Medicine, Hebron University, Hebron, Palestine

Dear Editor, Juvenile idiopathic arthritis (JIA) is the most common chronic rheumatic condition in children, with significant potential for long-term disability [1]. It encompasses several subtypes, including RF-negative polyarthritis, which may present with a rare form called dry synovitis (DS) [1, 2]. DS is characterized by progressive stiffness, functional limitation and radiologic damage without marked joint swelling or significant laboratory inflammation. Because of its subtle presentation, DS is often underdiagnosed, leading to delayed treatment and irreversible joint damage [2].

Here we describe the case of a young Palestinian child with RF-negative polyarticular JIA complicated by DS, a rare and challenging manifestation within the JIA spectrum. This case aims to raise awareness of DS, emphasize its diagnostic and therapeutic challenges and highlight the importance of early recognition, particularly in resource-limited settings.

A 6.5-year-old girl with a weight of 22 kg and height of 105 cm was referred to the paediatric rheumatology clinic following a diagnosis of uveitis at an ophthalmology clinic and a 3-month history of bilateral hand pain associated with morning stiffness lasting more than 1 h. She had no history of fever, rash, oral ulcers, diarrhoea, weight loss or constitutional symptoms. Her past medical history was notable for chronic abdominal pain, and she had been diagnosed with *Helicobacter pylori* infection 6 months earlier, for which she received standard triple therapy. Her family history was unremarkable for autoimmune diseases.

On initial physical examination, there was tenderness over the right 4th and 5th PIP joints, but no swelling, warmth, erythema or limitation in the range of motion. No other joints were involved and systemic examination was unremarkable.

Initial laboratory investigations revealed a normal ESR and unremarkable urinalysis. ANA was positive at a titre of 1:160 with a speckled pattern, while RF and anti-CCP antibodies were negative. Hand X-rays interpreted by a paediatric radiologist showed decreased joint space in the PIP joints and periarticular osteopenia of small joints in the hands and feet (Fig. 1).

**Figure 1:**
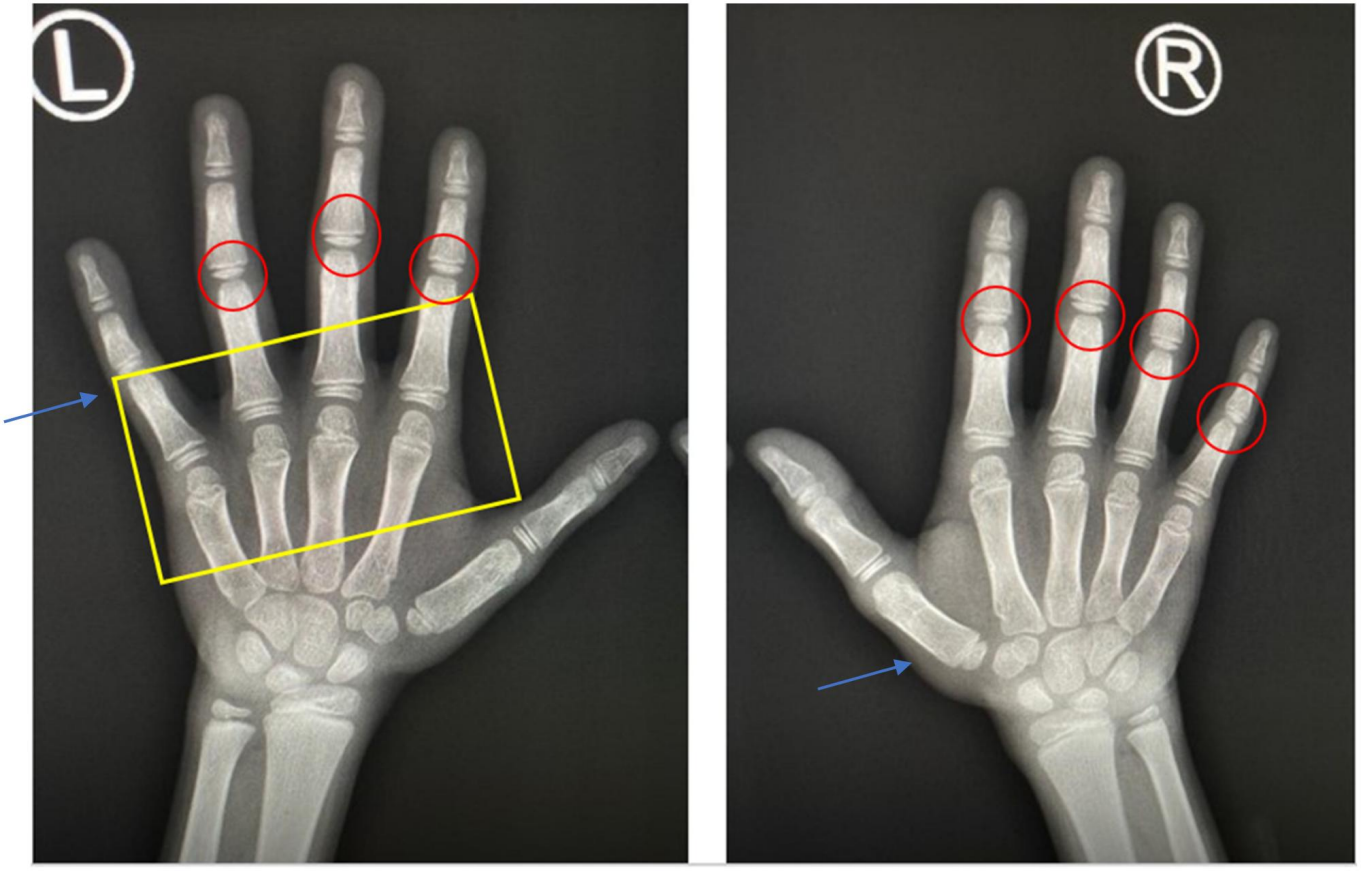
Initial bilateral hand radiographs of the patient. The red circles highlight joint space narrowing in the PIP joints bilaterally, while the yellow rectangle outlines areas of periarticular osteopenia, more prominent in the left MCP region. These findings, observed in the absence of overt joint swelling or effusion, are consistent with early radiologic features of DS

Based on these investigations, a provisional diagnosis of RF-negative polyarticular JIA was made. The patient was started on naproxen 250 mg twice daily and methotrexate 7.5 mg weekly, with folic acid supplementation. MTX was initiated at a conservative dose of 7.5 mg/week orally (≈9.3 mg/m^2^) after discussion within a multidisciplinary team. This decision was influenced by the lack of definitive inflammatory signs (no joint swelling, normal ESR/CRP) and the patient’s young age (estimated BSA ≈0.8 m^2^).

Three weeks later the patient came to our clinic complaining of worsening morning stiffness and new joint discomfort, with mild swelling in the knees, wrists and ankles. On physical examination, there was tenderness in the right wrist but still no overt signs of synovitis such as warmth, redness or restricted range of motion. Despite these symptoms, repeated laboratory tests remained normal, including ESR, CRP, complement levels (C3, C4) and complete blood count. Renal and liver function tests were also within normal limits.

Given the persistent symptoms in the absence of systemic inflammation or definitive joint swelling, the possibility of DS was strongly considered. Accordingly, the treatment plan was adjusted to include prednisone 5 mg daily, an increased methotrexate dose to 10 mg/week subcutaneously to improve bioavailability and continued folic acid. This regimen led to notable clinical improvement on follow-up.

On regular follow-up after 3 months, the patient was complaining of nausea, vomiting and epigastric discomfort, which are known side effects of MTX, in addition to a new attack in the context of right shoulder pain and swelling of her right wrist joint that lasted for 3 days without a history of fever or skin rash. Although the appropriate next step would have been to increase the MTX dose to 15 mg/m^2^, we instead switched to leflunomide 10 mg/day to avoid worsening side effects. This change was well tolerated and led to significant clinical improvement.

After 2 months of treatment with leflunomide, the patient showed very good clinical improvement, with resolution of joint symptoms and improved functional status. Physical examination was unremarkable and laboratory investigations remained within normal limits. Following this favourable response, continued treatment was maintained and at the 10-month follow-up (after 8 additional months) she remained clinically stable with no new complaints. At that point, in light of sustained remission, we decided to adjust the treatment plan, which included tapering and discontinuation of prednisone as well as discontinuation of both leflunomide and folic acid.

However, 1.5 years later she returned with a 1-month history of daily morning stiffness and joint pain involving the hips, shoulders, elbows and wrists. Examination revealed mild effusion in the right knee, with no other findings. Laboratory investigations remained unremarkable. Given the chronic relapsing course, absence of serologic inflammation, subtle radiologic findings and good response to immunosuppression, a final diagnosis of DS was established. MTX was reintroduced at 11 mg (≈14 mg/m^2^) weekly, along with folic acid 5 mg weekly, leading to sustained clinical improvement. She remains stable under follow-up.

DS is an uncommon and underrecognized variant of JIA characterized by minimal or no joint swelling, increasing stiffness and functional limitations [3, 4]. DS was first described by Levinson in 1974 as primarily associated with RF-negative polyarticular JIA, and its reported prevalence remains unclear due to limited documentation [5, 6]. The condition is frequently insidious, with delayed diagnosis due to mild clinical symptoms and the absence of overt inflammation [7].

Recognizing DS early is critical for preventing long-term joint damage and maintaining function. Delayed or missed diagnoses are common because DS does not usually manifest the classic signs of inflammation. This frequently ends in chronic disability, making it even more crucial for healthcare providers to be careful when evaluating patients with prolonged, unexplained joint stiffness, even if the typical indications of arthritis are absent in reported cases of DS, as ANA is typically negative [2]. However, there is little to no published evidence of ANA-positivity in DS itself, although ANA positivity is common in other JIA subtypes such as early-onset oligoarthritis [7, 8]. This variability highlights the need for a tailored diagnostic approach, as overlapping features may obscure the specific JIA subtype and complicate diagnosis.

Our patient’s clinical picture correlates with typical DS symptoms, which include increasing, symmetrical joint stiffness with limited or absent inflammation and joint swelling. While the upper limbs are frequently involved, our case showed lower limb stiffness and recurring episodes involving several joints. The child initially responded to NSAIDs and low-dose methotrexate but later relapsed and experienced gastrointestinal side effects, prompting a switch to leflunomide, which led to clinical improvement. Recurrence 1.5 years later reaffirmed the chronic nature of DS, which ultimately responded again to methotrexate. This case also highlights the need for individualized, symptom-guided management in atypical JIA variants like DS and underscores the limited evidence guiding therapy for this rare condition.

The diagnosis of DS is mainly clinical, supported by imaging and the exclusion of alternative causes. Proposed criteria include stiffness and painful joints for >3 months, limited or absent joint effusion, restricted range of motion (with or without contractures) and improvement with immunomodulatory therapy [5]. Our patient’s constant normal inflammatory markers, radiologic evidence of joint damage and absence of synovitis on physical examination all meet these criteria.

Radiological investigations are crucial for diagnosing and managing DS due to its subtle clinical features. A 2023 multicentre study by De Somer *et al.* [2] highlighted diagnostic challenges from the disease’s gradual onset. Conventional radiographs may be normal early on but later show joint space narrowing, juxta-articular osteopenia, bony erosions and growth disturbances, despite minimal swelling. MRI, especially with contrast, is the most sensitive for early detection, revealing synovial thickening, bone marrow oedema and little or no effusion, helping differentiate DS from other JIA subtypes. Ultrasonography can also detect subclinical synovial changes and monitor disease activity. In our patient, financial constraints limited MRI access, restricting early detailed assessment.

The differential diagnoses of DS include skeletal dysplasias like progressive pseudorheumatoid dysplasia, neuromuscular disorders, mucopolysaccharidoses and chromosomal disorders such as Turner syndrome [9–11]. These conditions were excluded in our patient based on the absence of systemic features, normal growth parameters and the chronic, progressive nature of DS, which distinguishes it from other inflammatory or systemic disorders.

This case highlights several important diagnostic limitations in the evaluation of DS, particularly in resource-limited settings. The absence of classic inflammatory features—such as joint swelling, elevated inflammatory markers or systemic symptoms—contributed to delayed clinical suspicion. Furthermore, advanced imaging modalities like MRI and musculoskeletal ultrasound, which are essential for early detection of synovial inflammation and joint damage, were not accessible due to financial constraints. Instead, reliance on conventional radiographs, which may be normal in the early disease stages, increased the risk of misclassification and diagnostic delay. Additionally, the lack of universally accepted diagnostic criteria for DS further complicates its differentiation from other JIA subtypes or skeletal dysplasias. These limitations emphasize the need for increased clinical awareness, improved access to sensitive diagnostic tools and the development of standardized diagnostic pathways, especially in underresourced healthcare environments.

In conclusion, DS should be included in the differential diagnosis of children with chronic joint stiffness and, with minimal inflammation findings, especially among RF-negative JIA. This case highlights the importance of high clinical suspicion, imaging and flexibility in the treatment approach. It also highlights the need to establish uniform diagnostic criteria and evidence-based management guidelines for this rare and much-overlooked JIA subgroup.

## Data Availability

All data generated or analysed during this study are included in this published article. Additional clinical details can be provided by the corresponding author upon reasonable request, respecting patient confidentiality and in compliance with applicable ethical regulations.
